# Nonsurgical Retreatment of Endodontically Treated Teeth: A Prospective Cohort Study on Success Rates and Quality of Life

**DOI:** 10.7759/cureus.88886

**Published:** 2025-07-28

**Authors:** Megna Bhatt, Tanya Seth, Swati Chaudhary, Ketaki Rajguru, Chaitanya S Khalane, Adhishree S Chib, Seema Gupta

**Affiliations:** 1 Department of Conservative Dentistry and Endodontics, Shree Bankey Bihari Dental College and Research Centre, Ghaziabad, IND; 2 Department of Conservative Dentistry and Endodontics, Tatyasaheb Kore Dental College and Research Centre, Nave Pargaon, IND; 3 Department of Oral Pathology, Jawahar Medical Foundation's Annasaheb Chudaman Patil Memorial Dental College, Dhule, IND; 4 Department of Pediatric Dentistry, Muskaan Dentals Global, Gurugram, IND; 5 Department of Orthodontics, Kothiwal Dental College and Research Centre, Moradabad, IND

**Keywords:** endodontic, quality of life, retreatment, root canal therapy, success

## Abstract

Introduction: The present study evaluated the clinical and patient-centered outcomes of nonsurgical retreatment of endodontically treated teeth. The primary goal was to assess the success rate, defined by clinical and radiographic criteria, while the secondary objectives focused on patient-reported quality-of-life outcomes using a validated oral health-related quality-of-life (OHRQoL) questionnaire.

Materials and methods: This prospective cohort study was conducted from January 2024 to April 2025 at the Department of Conservative Dentistry and Endodontics and included 100 systemically healthy adults (aged 18-60 years) requiring retreatment of endodontically treated teeth. Patients with vertical root fractures, advanced periodontal disease, or non-restorable teeth were excluded. The procedure was performed using current/contemporary endodontic technologies such as rotary files, ultrasonic irrigation, and warm vertical compaction with gutta-percha and root canal sealer. Calcium hydroxide was used as an intracanal medication between visits. Outcomes were assessed six months after permanent restoration via clinical examinations and digital periapical radiographs, categorizing cases as healed, healing, or non-healing. OHRQoL scores were collected preoperatively and one- and six-months post-treatment using a modified questionnaire with a 1-5 Likert scale. Statistical analyses included Friedman’s test for OHRQoL scores and Fisher’s exact test for success rates with a significance level of p < 0.05.

Results: Of 100 patients, 88 (88%) patients achieved successful outcomes (healed, healing, or non-healing). Maxillary jaw retreatments outperformed mandibular jaw retreatments, and cases with periapical lesions had higher success rates than those without periapical lesions. No demographic or clinical factor significantly influenced the success rate (p > 0.05). Successful cases showed significant OHRQoL improvement (p = 0.001) from baseline (32.13 ± 12.07) to six months (19.40 ± 12.07), while failed cases showed no significant change (p = 0.425).

Conclusion: Nonsurgical root canal retreatment in endodontically treated teeth achieved a high success rate with significant quality-of-life improvements in successful cases, underscoring the efficacy of standardized protocols and advanced technologies.

## Introduction

Nonsurgical root canal retreatment is a critical procedure in endodontics aimed at addressing failed initial root canal treatments, which may present with persistent symptoms or radiographic evidence of periapical pathology [1]. Despite the high success rates of root canal treatment (RCT), reported to be 97% at 10 years of follow-up and 76% after 30 years, failures can occur, necessitating interventions such as retreatment or apical surgery [2]. The most common causes of failed RCTs are inappropriate chemo-mechanical debridement, persistence of bacteria in the canals, poor obturation quality, over- and under-extension of the root canal filling, and coronal leakage [3]. Previous meta-analyses have indicated that nonsurgical retreatment achieves a pooled success rate ranging from 76.7% to 77.8%, with variability attributed to factors such as preoperative periapical status, lesion size, and coronal restoration quality [4,5].

Nonsurgical root canal retreatment is indicated for persistent symptoms like pain or sensitivity, periapical radiolucency showing treatment failure, incomplete initial treatment with missed canals or poor obturation, coronal leakage, or reinfection by bacteria such as *Enterococcus faecalis* in restorable teeth. It is contraindicated in cases of vertical root fractures, advanced periodontal disease, non-restorable teeth, or major malocclusion [4,5]. Advancements in root canal instrumentation have transformed endodontics, shifting from manual to mechanical and rotary techniques using nickel-titanium (NiTi) alloys. These innovations enhance procedural accuracy, improve treatment efficacy, and reduce patient discomfort [6]. However, their impact on clinical outcomes remains underexplored, particularly in prospective studies focusing on specific tooth types, such as the first molars, which are critical for masticatory function.

The integration of patient-centered outcomes with traditional clinical and radiographic metrics is increasingly recognized as essential for the comprehensive evaluation of treatment efficacy [7]. Oral health-related quality of life (OHRQoL) encompasses functional, psychological, and social dimensions that may be influenced by dental conditions and their treatment [8]. Similarly, subjective chewing ability serves as a practical measure of restored dental function, especially for posterior teeth, such as the first molars, which play a pivotal role in mastication [9]. A previous study conducted by He et al. [10] demonstrated that nonsurgical retreatment using contemporary techniques can achieve a success rate of 90.4% in first molars while significantly improving OHRQoL and chewing ability. 

This study aimed to evaluate the clinical and patient-centered outcomes of nonsurgical root canal retreatment in endodontically treated teeth using a standardized protocol incorporating advanced endodontic technologies. The primary objective was to determine the success rate of retreatment, defined by clinical and radiographic criteria, by categorizing the outcomes as healed, healing, or nonhealing. The secondary objective was to assess patient-centered outcomes using validated OHRQoL to capture the impact of treatment on patients’ quality of life and functional restoration.

## Materials and methods

Study design and setting

This prospective cohort study was conducted at the Department of Conservative Dentistry and Endodontics, Shree Bankey Bihari Dental College and Research Centre, Ghaziabad, India, between January 2024 and April 2025. The research protocol was approved by the Institutional Ethical Committee (SBBDC/2023/115). Written informed consent was obtained from all patients prior to enrollment, and the study followed the principles of the Declaration of Helsinki.

Patient population

Patients referred to the endodontic clinic for the retreatment of permanent teeth were recruited. The inclusion criteria were as follows: adult patients aged 18-60 years; systemically healthy patients without immunocompromising conditions (such as uncontrolled diabetes and acquired immunodeficiency syndrome), classified as ASA I or ASA II per the American Society of Anesthesiologists (ASA) Physical Status Classification System; and previously endodontically treated maxillary or mandibular anterior or posterior teeth with opposing dentition that required retreatment. The exclusion criteria were vertical root fracture, advanced periodontal disease, non-restorable teeth, and major malocclusion.

Restorability was determined by consensus between supervising endodontic and restorative faculties. Preoperative diagnoses were established based on clinical and radiographic findings using the American Association of Endodontists Consensus Conference-recommended terminology, categorizing pulpal status as previously treated and periapical status as normal, symptomatic apical periodontitis, asymptomatic apical periodontitis, or chronic apical abscess [11].

Sample size calculation

The sample size for this study was calculated using G*Power 3.1 software (Heinrich-Heine-Universität Düsseldorf, Düsseldorf, Germany) based on data from prior research showing an odds ratio of 2.23 for treatment success in retreated teeth with periapical lesions [10]. With 80% power (β = 0.20) and 5% two-tailed alpha error (α = 0.05), the calculation required 90 patients. To account for potential attrition of approximately 10%, the target sample size was increased to 100 patients.

Treatment protocol

Retreatment procedures were performed by trained endodontists using a standardized protocol (Adhishree Chib, Tanya Seth, and Megna Bhatt). The patients were anesthetized using 2% lidocaine with 1:100,000 epinephrine (Xylocaine, Dentsply Sirona, Charlotte, NC, USA) and isolated using a rubber dam (Hygienic Dental Dam, Coltene/Whaledent, Cuyahoga Falls, OH, USA). The caries and defective restorations were removed, and a re-access was prepared using high-speed carbide burs (SS White Dental, Lakewood, NJ, USA) to achieve straight-line access.

Obturational materials and obstructions were removed using a combination of heat (System B, Kerr Dental, Orange, CA, USA), solvent (EndoSolv, Septodont, Saint-Maur-des-Fossés, France), hand files (K-Files, Dentsply Sirona, Charlotte, NC, USA), rotary files (ProTaper Next, Dentsply Sirona, Charlotte, NC, USA), and ultrasonic instruments (Satelec P5 Newtron, Acteon North America, Mount Laurel, NJ, USA). Working length was determined using an electronic apex locator (Root ZX II, J. Morita, Irvine, CA, USA) and confirmed using digital radiographs (Schick 33, Dentsply Sirona, Charlotte, NC, USA).

Root canal instrumentation was performed using a crown-down technique with K-Files (sizes #10-25) and ProTaper Next rotary files (X1-X4, corresponding to tip sizes #17-40 with 0.04-0.06 taper, Dentsply Sirona, Charlotte, NC, USA), with irrigation using 20 mL 5.25% sodium hypochlorite (NaOCl, Clorox, Oakland, CA, USA) and 5 mL 17% ethylenediaminetetraacetic acid (EDTA, Pulpdent, Watertown, MA, USA). Mesial and buccal canals were prepared to an apical size of #35-40 with a 0.04 or 0.06 taper; distal and palatal canals were prepared to #40-60 with a 0.04 or 0.06 taper, depending on canal anatomy. Passive ultrasonic irrigation with NaOCl was performed for 15 seconds per canal using a #15 stainless steel file with an NSK Varios 970 ultrasonic unit (NSK America, Hoffman Estates, IL, USA). Surgical operating microscopes were employed to improve visualization and accuracy during retreatment procedures.

Treatments were completed in two to three visits. An intracanal dressing of calcium hydroxide (UltraCal XS, Ultradent, South Jordan, UT, USA) was placed for 1-2 weeks between visits, with an intermediate restorative material (IRM; Dentsply Sirona, Charlotte, NC, USA) used as the interim filling material. During the obturation visit, calcium hydroxide was removed using 5.25% NaOCl and ProTaper Next rotary instrumentation. Passive ultrasonic irrigation with NaOCl was performed to ensure the thorough removal of the medication. The smear layer was removed using 5 mL of 17% EDTA. The canals were dried using paper points (Dentsply Sirona, Charlotte, NC, USA) and obturated with gutta-percha (Dentsply Sirona) and AH Plus sealer (Dentsply Sirona) using the warm vertical compaction technique with System B (Kerr Dental, Orange, CA, USA) and Obtura III (Obtura Spartan, Algonquin, IL, USA). An IRM was used for temporary restoration. 

Permanent coronal restorations were performed by the same endodontists who conducted the retreatment, ensuring continuity and standardization of care. Full-coverage crowns, either porcelain-fused-to-metal or all-ceramic, were placed within one month post-obturation to provide a robust coronal seal and protect the tooth structure from fracture or reinfection. Crowns were cemented using resin-modified glass ionomer cement (RelyX Luting, 3M ESPE, St. Paul, MN, USA), and their integrity was verified clinically and radiographically to ensure a tight marginal fit.

Outcome assessment

The patients were recalled six months after permanent coronal restoration placement. Clinical examinations assessed coronal restoration integrity, pain or discomfort on palpation, percussion, biting (using the Tooth Slooth, Professional Results Inc., Laguna Niguel, CA, USA), periodontal probing depths, and sinus tract presence. Digital periapical radiographs (Schick 33, Dentsply Sirona, Charlotte, NC, USA) were evaluated by three calibrated observers (Swati Chaudhary, Ketaki Rajguru, and Chaitanya Khalane) to determine the periapical status. Outcomes were classified as follows: healed with no clinical signs or symptoms; normal periapical tissues with intact periodontal ligament space or slightly widened ligament around the extruded material; healing with no clinical signs or symptoms; periapical radiolucency present but reduced in size; non-healing with the presence of signs or symptoms; new periapical radiolucency; or unchanged/enlarged radiolucency. Non-healing was considered a failure, while healing was considered successful [10]. 

A novel OHRQoL questionnaire was developed for this study (provided in appendices) to assess patient outcomes at preoperative and one- and six-month follow-up visits following root canal retreatment in first molars. The questionnaire was designed based on established constructs from prior literature [10], focusing on domains such as pain, chewing function, aesthetics, psychological impact, and social interactions. To ensure relevance to the study population, an interdisciplinary team of endodontists and public health researchers drafted 20 original items using patient-centered language, avoiding direct replication of existing tools. Content validity was established through expert review by three independent dental specialists, who evaluated item clarity, relevance, and alignment with OHRQoL constructs, resulting in minor revisions to two items. The questionnaire was pilot tested with a sample of 30 patients undergoing root canal retreatment at the department. Reliability was assessed using Cronbach’s alpha, yielding an internal consistency of 0.82, indicating good reliability. Test-retest reliability was evaluated in a subset of 15 patients over a two-week interval, with an intraclass correlation coefficient (ICC) of 0.78 (95% CI: 0.65-0.89), demonstrating stable responses. The final questionnaire comprised 20 items with responses recorded as yes or no.

Statistical analysis

The data were entered into Microsoft Excel (Redmond, USA), and all statistical analyses were performed by using the IBM Corp. Released 2014. IBM SPSS Statistics for Windows, Version 23.0. Armonk, NY: IBM Corp. The distribution of continuous data was checked using the Shapiro-Wilk test and proved to be non-normally distributed; therefore, a non-parametric test (Friedman test) was used to compare the OHRQoL scores. Categorical data are presented as frequencies, and Fisher’s exact test was used to analyze the success rate of endodontic retreatment with different parameters. The level of significance was kept at a p-value < 0.05.

## Results

Demographic characteristics of patients are presented in Table 1. The sample consisted of 100 patients, with a slightly higher proportion of males than of females. The mean age of males was 43.56 ± 4.52 years, and the mean age of females was 42.89 ± 3.12 years. Regarding jaw distribution, a near-equal representation was observed, in which retreatments were required slightly more frequently in the upper jaw than in the lower jaw. Preoperative diagnoses varied, with asymptomatic apical periodontitis and symptomatic apical periodontitis being the most common, followed by chronic periapical abscesses and normal cases. Posterior teeth were more frequently involved than the anterior teeth (Table 1).

**Table 1:** Demographic characteristics of the sample. Data are presented as frequency (n) and percentage (%), where n denotes the number of patients.

Variables	Category	n	%
Sex	Female	42	41.17
	Male	58	56.86
Jaw	Mandibular	48	47.05
	Maxillary	52	50.98
Preoperative diagnosis	Asymptomatic apical periodontitis	32	31.37
	Chronic periapical abscess	20	19.60
	Normal endodontically treated teeth	18	17.64
	Symptomatic apical periodontitis	30	29.41
Site	Anterior teeth	40	39.21
	Posterior teeth	60	58.82

The success rates of the retreated endodontic cases were analyzed based on various demographic and clinical factors. Females had an 86% success rate compared to 90% in males, and the odds ratio (OR = 0.18) suggested that females had a slightly lower likelihood of success, but this was not a strong predictor. Maxillary jaw retreatment had a higher success rate (92%) than mandibular jaw retreatment (83%). Patients without preoperative pain showed an 84% success rate, whereas those with pain had a 92% success rate. Interestingly, patients with periapical lesions had a higher success rate (91%) than those without (81%). Smaller lesions (< 5 mm) had a marginally higher success rate (94%) than larger lesions (> 5 mm, 89%). Anterior teeth had a 90% success rate compared to 87% for posterior teeth. Although some trends were observed (such as higher success with maxillary jaw retreatment and absence of pain), none of the variables showed a statistically significant association with success rates (p > 0.05). This suggested that factors such as sex, jaw location, pain, and lesion status did not strongly influence the outcomes of endodontic retreatment in this study (Table 2).

**Table 2:** Odds ratio for success rate of retreated cases. A p-value > 0.05 denotes statistical non-significance.

Variables	Categories	Total	Success	Failures	Success rate	p-value	Odds ratio
Sex	Female	42	36	8	86%	0.674	0.18
	Male	58	52	6	90%		
Jaw	Maxillary	48	40	8	83%	0.329	0.95
	Mandibular	42	48	4	92%		
Pain	Absent	50	42	8	84%	0.384	0.75
	Present	50	46	4	92%		
Periapical lesion	Absent	32	26	6	81%	0.314	1.01
	Present	68	62	6	91%		
Size of lesion	< 5 mm	32	30	2	94%	0.542	1.21
	> 5 mm	36	32	4	89%		
Site	Anterior	40	36	4	90%	0.722	0.13
	Posterior	60	52	8	87%		

Out of 100 patients, 88 (88%) showed success. The Friedman test was used to analyze changes in OHRQoL scores over time. At baseline, patients with failed retreatments had significantly higher (worse) OHRQoL scores (mean = 49.83 ± 4.75) than those with successful retreatments (mean = 32.13 ± 12.07). Over time, the failed cases showed a slight worsening in OHRQoL scores at one month (51.16 ± 3.48) and six months (52.53 ± 4.76), but these changes were not statistically significant (p = 0.425). In contrast, successful cases demonstrated a significant improvement (p = 0.001) in OHRQoL scores, decreasing from 32.13 (baseline) to 24.38 (one month) and further to 19.40 (six months). The findings indicated that successful endodontic retreatment led to a progressive and statistically significant improvement in patients' OHRQoL over six months. In contrast, failed retreatment did not result in meaningful changes in OHRQoL, with scores remaining consistently poor. This finding suggested that treatment success was strongly associated with better long-term patient-reported outcomes (Table 3).

**Table 3:** Friedman analysis for repeated measurement of oral health-related quality of life (OHRQoL) scores. *p-value < 0.05 denotes statistical significance. Data are presented as mean and standard deviation (SD); n denotes the number of patients.

Response	n (%)	OHRQoL (Mean ± SD)			F value	p-value
		Baseline	One-month	Six-month		
Failure	12 (12)	49.83 ± 4.75	51.16 ± 3.48	52.53 ± 4.76	1.56	0.425
Success	88 (88)	32.13 ± 12.07	24.38 ± 9.75	19.40 ± 8.18	5.34	0.001*

Post-hoc analysis (Conover test) revealed significant OHRQoL score improvements in successful retreatments across all intervals (p < 0.001). The largest difference occurred between baseline and six months (mean difference = 12.73), followed by baseline vs. one month (7.75) and one month vs. six months (4.98). This demonstrated a progressive, statistically significant quality-of-life enhancement postoperatively (Table 4).

**Table 4:** Post-hoc analysis using the Conover test with Bonferroni correction for successful cases. *p-value < 0.05 denotes statistical significance.

Pairwise comparison	Mean difference	T Stats	p-value
Baseline vs. one-month	7.75	9.49	0.001*
Baseline vs. six-month	12.73	20.55	0.001*
One-month vs. six-month	4.98	11.05	0.001*

## Discussion

This prospective cohort study evaluated the clinical and patient-centered outcomes of nonsurgical root canal retreatment in first molars using a standardized protocol with contemporary endodontic tools. The study achieved a success rate of 88% (combining healed and healing cases), with 92% of the upper jaw and 83% of the lower jaw retreatments classified as successful. Additionally, significant improvements in OHRQoL were observed in successful cases, particularly within the first six months postoperative, highlighting the positive impact of retreatment on patients’ functional and psychological well-being.

The success rate of 88% aligns with but is slightly lower than the 90.4% reported by He et al. [10] in a similar prospective study of first molar retreatment. This difference may be attributed to variations in sample size and follow-up duration. Notably, this study did not employ cone beam computed tomography (CBCT) but instead relied on digital periapical radiographs, which is consistent with the methodology of previous studies [10,12]. According to Elemam and Pretty [13], most studies conducted so far have considered multi-rooted teeth, with only one study taking single-rooted teeth into consideration. However, in the present study, both multi-rooted and single-rooted teeth were considered. According to a previous systematic review, the variability in research design, sample dimensions, patient demographics, therapeutic regimens, and methodological approaches across the studies assessing the outcomes of nonsurgical retreatment of endodontically treated teeth might constrain the comparability and generalizability of the findings [14].

The standardized protocol, incorporating tools such as surgical operating microscopes, ultrasonic irrigation, and rotary instrumentation, likely enhanced the technical precision of retreatment [15]. These tools facilitate the removal of previous obturation materials, identification of missed canals, and effective disinfection, addressing common causes of initial treatment failure such as intraradicular infection or incomplete cleaning [15]. The use of 1% NaOCl with passive ultrasonic irrigation likely improved canal cleanliness against *Escherichia coli* and *Streptococcus mutans* [16].

The findings of the present study indicated a lack of statistically significant differences in success rates across variables such as sex, jaw location, preoperative pain, lesion status, and tooth type, suggesting that these factors may not be strong predictors of outcome in this cohort, possibly because of the limited sample size, reducing statistical power, as noted by He et al. [10]. The higher success rate (91%) in cases with preoperative periapical lesions compared to those without (81%) in our study contrasts with previous findings, where poorer outcomes with lesions due to factors such as resistant infections or larger lesion size were reported [5]. This discrepancy may stem from our standardized protocol using advanced tools such as surgical microscopes, ultrasonic irrigation, and rotary files, which effectively managed infections with 5.25% NaOCl and 17% EDTA. The six-month follow-up, shorter than the 24 months in He et al. [10], likely captured early healing, inflating success rates for lesion cases, while the absence of CBCT imaging may have underestimated pathology in “no lesion” cases. A potentially less severe lesion cohort (smaller lesions had a 94% success rate vs. 89% for larger lesions) and limited statistical power (n = 100, p > 0.05) may further explain this trend, suggesting that our findings may not robustly challenge the established literature without longer follow-up or larger samples.

The significant improvement in OHRQoL scores among successful cases (from 32.13 at baseline to 19.40 at six months, p = 0.001) underscores the patient-centered benefits of retreatment. The progressive improvement, with the largest change between baseline and six months (mean difference = 12.73), aligns with the findings of He et al. [10], where the greatest OHRQoL and chewing ability improvements occurred within the first week post-treatment, with continued gains over time. In contrast, failed cases showed persistently poor OHRQoL scores (mean 52.53 at six months; p = 0.425), indicating that unsuccessful retreatment did not alleviate the functional or psychological burden of persistent pathology. These findings emphasize the importance of patient-centered outcomes, such as OHRQoL, in assessing treatment efficacy, as they capture dimensions such as pain, chewing ability, and psychological comfort, which are critical to patients’ overall well-being [17,18].

The absence of significant differences in the success rates between the maxillary (92%) and mandibular (83%) molars contrasts with He et al. [10], where maxillary molars showed a higher healing rate (88.2% vs. 62.9%), although the difference was not statistically significant. This discrepancy may reflect differences in operator experience or canal anatomy challenges, although the standardized protocol in this study likely mitigated such variability.

There exist numerous obstacles encountered during the process of retreatment, which encompass the extraction of the prior obturation material, rectifying procedural errors that occurred during the initial intervention, identifying overlooked canals, and eradicating potentially therapy-resistant bacteria. The prognosis is typically diminished in retreatment as opposed to the initial treatment due to these challenges [10]. Therefore, the primary objectives of non-surgical retreatment are the total extraction of gutta-percha from the walls of the root canal, the reinstatement of working length, the facilitation of disinfection, and the subsequent re-obturation of the root canal, all aimed at restoring healthy periapical tissues and achieving a reliable outcome [19]. According to Kasam and Mariswamy [20], the ultrasonic retreatment tip was an efficient method to remove gutta-percha. In scenarios where the original anatomical configuration was modified due to the primary orthograde intervention, the success rate of retreatments at the two-year mark was significantly reduced by 50% (from 80-90% to 40-50%), irrespective of the type of tooth involved [21]. 

This prospective cohort study achieved an 88% success rate in nonsurgical root canal retreatment of teeth and highlighted the effectiveness of a standardized protocol using contemporary endodontic techniques to enhance clinical outcomes and OHRQoL. To ensure the success of the initial RCT and prevent the need for retreatment, clinicians should prioritize thorough canal disinfection using 1% NaOCl with passive ultrasonic irrigation to eliminate microbial biofilms [16]. Identifying all canals, especially in complex first molars, is critical and requires surgical operating microscopes and adequate access preparation with high-speed carbide burs. CBCT should be used for initial diagnosis and treatment planning [22]. Bhatt et al. [22] conducted an investigation into the influence of CBCT on clinical decision-making and initial diagnostic processes, specifically in relation to conventional radiographic methods concerning endodontic therapies. Their findings indicated that the supplementary data acquired from CBCT imaging led to alterations in the preliminary diagnoses and, consequently, the associated treatment regimens in 59 out of 96 root canal retreatment cases (61%) and in 64 out of 96 root canal retreatment cases (66%).

The etiological and pathological origins of endodontic infections are predominantly bacterial in nature; therefore, the elimination of microorganisms and their metabolic by-products from the root canal system is imperative to achieve a favorable long-term prognosis. During retreatment, meticulous care is essential to address challenges such as persistent infections and procedural errors from prior treatments. Clinicians should employ a systematic approach to remove previous obturation materials. Thorough disinfection is crucial for eliminating resistant bacteria, as intra-radicular infection is the most common cause of RCT failure [3]. A previous study has reported a variety of microorganisms, including cocci, rods, and filamentous forms, in root canal-treated teeth with apical periodontitis [23]. Careful assessment of tooth restorability, avoidance of iatrogenic errors, such as perforations, and ensuring high-quality coronal restoration after retreatment are vital for long-term success [15]. Clinicians should also consider patient-centered outcomes, as significant OHRQoL improvements in successful cases emphasize the importance of restoring function and comfort.

Limitations of this study included the relatively short six-month follow-up period, which might not capture long-term healing trends, as some lesions might require up to 24 months to fully resolve. Additionally, a sample size of 100 might still lack sufficient power to detect significant differences in prognostic factors such as preoperative pain or lesion size. The absence of CBCT imaging might have underestimated periapical pathology, potentially inflating the success rate. Finally, the use of only gutta-percha with AH Plus sealer eliminated a potential variable but precluded the comparison of the impact of obturation materials.

Future research should incorporate longer follow-up periods to assess sustained healing and include CBCT imaging to enhance diagnostic accuracy. Larger sample sizes and randomized controlled trials could further elucidate the impact of specific techniques or preoperative factors on outcomes. Additionally, exploring the role of emerging technologies, such as laser-assisted irrigation and regenerative endodontic approaches, could provide insights into further improving the retreatment success.

## Conclusions

This prospective cohort study demonstrated that nonsurgical root canal retreatment of teeth using a standardized protocol with contemporary endodontic techniques achieved an 88% success rate (combining healed and healing cases) at a six-month follow-up, with higher success in the maxillary teeth compared to the mandibular teeth. Significant improvements in OHRQoL were observed in successful cases, highlighting the positive impact of treatment on patients’ functional and psychological well-being. The higher success rate in patients with preoperative periapical lesions suggests that thorough disinfection and advanced methods, including ultrasonic irrigation and rotary instrumentation, effectively address persistent infections. These findings affirmed the efficacy of nonsurgical retreatment in restoring tooth function and quality of life, supporting its role as a viable option for managing failed endodontic treatments, although longer follow-up studies are needed to confirm the sustained outcomes.

## Appendices

Questionnaire

Part 1: Demographic Questions

Name:

Age:

Sex:

Dental visits in the last 6 months:

1

2

3

>3

Dental treatment in the last 6 months:

Scaling and polishing

Cavity filling

Root canal

Any other

Root canal treatment (Duration)-

In the last 1 month

In the last 6 months

In the last 1 year

Number of sitting in the root canal treatment:

1

2

3

>3

Part 2: Questions for Quality of Life

**Table 5:** Questionnaire (part 2) for quality of life. The oral health-related quality-of-life (OHRQoL) questionnaire was designed and tested by the principal investigator. Credit: Megna Bhatt and Tanya Seth.

S.no	Questions	Yes	No
1	Do you experience difficulty speaking clearly due to issues with your teeth or mouth?		
2	Have you noticed a decline in your ability to taste food because of your teeth or mouth?		
3	Do you suffer from discomfort or pain in your mouth?		
4	Is it challenging to eat certain foods comfortably due to your teeth or mouth?		
5	Have you needed to adjust the temperature of your food because of your teeth or mouth?		
6	Do you feel self-aware or uneasy about the appearance of your teeth or mouth?		
7	Have your teeth or mouth caused you to feel anxious or stressed?		
8	Does your diet feel inadequate due to problems with your teeth or mouth?		
9	Have you had to pause meals because of issues with your teeth or mouth?		
10	Is it hard to unwind or stay calm because of your teeth or mouth?		
11	Do you struggle to fall asleep due to discomfort from your teeth or mouth?		
12	Have problems with your teeth or mouth woken you up during the night?		
13	Do you feel ashamed or self-conscious about your teeth or mouth?		
14	Have your teeth or mouth made you feel frustrated with others?		
15	Does it interfere with your ability to perform daily tasks due to teeth or mouth issues?		
16	Have your teeth or mouth made you feel less content with life overall?		
17	Do you ever feel completely unable to carry out activities because of your teeth or mouth?		
18	Have you avoided certain social situations due to concerns about your teeth or mouth?		
19	Has the condition of your teeth or mouth affected your confidence in daily interactions?		
20	Do you find it necessary to modify your eating habits frequently because of your teeth or mouth?		
